# How Well Does the Standardized Video Interview Score Correlate with Traditional Interview Performance?

**DOI:** 10.5811/westjem.2019.7.42731

**Published:** 2019-08-20

**Authors:** Arlene S. Chung, Kaushal H. Shah, Michael Bond, Brahim Ardolic, Abbas Husain, Ida Li, Lukasz Cygan, William Caputo, Jan Shoenberger, Jeff van Dermark, Jonathan Bronner, Moshe Weizberg

**Affiliations:** * Maimonides Medical Center, Department of Emergency Medicine, Brooklyn, New York; † Icahn School of Medicine at Mount Sinai, Department of Emergency Medicine, New York City, New York; ‡ University of Maryland School of Medicine, Department of Emergency Medicine, Baltimore City, Maryland; § Staten Island University Hospital-Northwell Health, Department of Emergency Medicine, Staten Island, New York; ¶ Methodist Hospital, Department of Emergency Medicine, Brooklyn, New York; || Keck School of Medicine of the University of California, Department of Emergency Medicine, Los Angeles, California; # University of Texas Southwestern Medical Center, Department of Emergency Medicine, Dallas, Texas; ** University of Kentucky College of Medicine, Department of Emergency Medicine, Lexington, Kentucky

## Abstract

**Introduction:**

In 2017, all medical students applying for residency in emergency medicine (EM) were required to participate in the Standardized Video Interview (SVI). The SVI is a video-recorded, uni-directional interview consisting of six questions designed to assess interpersonal and communication skills and professionalism. It is unclear whether this simulated interview is an accurate representation of an applicant’s competencies that are often evaluated during the in-person interview.

**Objective:**

The goal of this study was to determine whether the SVI score correlates with a traditional in-person interview score.

**Methods:**

Six geographically and demographically diverse EM residency programs accredited by the Accreditation Council for Graduate Medical Education participated in this prospective observational study. Common demographic data for each applicant were obtained through an Electronic Residency Application Service export function prior to the start of any scheduled traditional interviews (TI). On each TI day, one interviewer blinded to all applicant data, including SVI score, rated the applicant on a five-point scale. A convenience sample of applicants was enrolled based on random assignment to the blinded interviewer. We studied the correlation between SVI score and TI score.

**Results:**

We included 321 unique applicants in the final analysis. Linear regression analysis of the SVI score against the TI score demonstrated a small positive linear correlation with an r coefficient of +0.13 (p=0.02). This correlation remained across all SVI score subgroups (p = 0.03).

**Conclusion:**

Our study suggests that there is a small positive linear correlation between the SVI score and performance during the TI.

## INTRODUCTION

The screening, interviewing, and ranking processes for residency programs are critical and have enduring consequences for the overall program. Residency leadership is tasked with identifying applicants who are a “good fit” for the program and have both a high likelihood of success and low likelihood of poor performance. This can be challenging when faced with applications that number in the hundreds to thousands in a typical application cycle. Traditional interviews (TI) are designed to assess for noncognitive factors, such as interpersonal and communication skills, maturity, interest in the field, dependability, and honesty, which cannot be easily assessed through other means.^1^

In 2017, the Association of American Medical Colleges (AAMC) required all medical students applying for residency in emergency medicine (EM) to complete the Standardized Video Interview (SVI) as part of the application process. The ultimate goal is to extend this to other specialties as well. The SVI is a recorded, asynchronous, and uni-directional video interview that consists of six questions presented in text prompts. Students have 30 seconds to read each question and up to three minutes to record a response. Each response is rated on a five-point scale that ranges from 1 = rudimentary to 5 = exemplary and the total score is calculated as the sum of the ratings from each response for a total score range of 6–30.^2^ Residency programs may view each applicant’s total score and also the entire video response of all six questions.

The SVI is designed to assess (1) interpersonal and communication skills, and (2) knowledge of professional behaviors.^2^ Previously, these two competencies could only be indirectly measured through personal statements, standardized letters of evaluation (SLOE), and selected quotes from each applicant’s medical student performance evaluation.^3^–^5^ Although the AAMC explicitly states that the SVI “is not intended to replace in-person interviews,”^2^ we sought to determine whether there is any correlation between the SVI and the TI. Given the large volume of applicants to each residency program, it is possible that some programs may use the SVI as a proxy measure of an applicant’s competencies that are often evaluated during the in-person interview. However, it is unclear if this simulated interview format is an accurate representation of an applicant’s relevant competencies. The goal of this study was to determine how well (if at all) the SVI score correlates with an in-person TI.

## METHODS

This was a prospective, observational, multicenter study conducted from October 2017–February 2018. Six EM residency programs accredited by the Accreditation Council for Graduate Medical Education (ACGME) participated in the study. Common demographic data for each applicant (gender, age, and United States Medical Licensing Exam score) were obtained through an Electronic Residency Application Service (ERAS) export function prior to the start of any scheduled TIs. During each TI day, one interviewer at each site was blinded to all applicant data, including the SVI score. This blinded interviewer met the applicant with no previous information regarding that applicant. The blinded interviewer was then asked to rate the TI on a five-point Likert scale (1 = rudimentary; 2 = below average; 3 = average; 4 = above average; 5 = exemplary) that was developed a priori through consensus by the authors. The scale was deemed to have face validity based on review by multiple residency program directors involved in this study. The blinded interviewer based his or her TI score purely on the interview. When a single applicant was interviewed at more than one program participating in this study, the mean TI score was used.

**Population Health Research Capsule**

What do we already know about this issue?

*The Standardized Video Interview (SVI) is a uni-directional video interview with six questions that assess interpersonal and communication skills and professionalism*.

What was the research question?

*Our goal was to determine whether the SVI score correlates with a traditional in-person interview (TI) score*.

What was the major finding of the study?

*The SVI score demonstrated a small, positive linear correlation with the TI score that remained across all SVI score subgroups*.

How does this improve population health?

*While the SVI may provide an estimate of an applicant’s performance on a TI, it may not be a true replacement for a traditional interview*.

A convenience sample of applicants was enrolled based on random assignment to the blinded interviewer. Inclusion criteria were applicants assigned to the blinded interviewer at a participating site. Exclusion criteria included prior knowledge of the applicant by the interviewer and no SVI score available for the applicant. We studied the correlation between SVI score and TI score. Predetermined subgroup analysis was performed based on applicants’ SVI scores as follows: 6–11, 12–17, 18–23, 24–30. These SVI score ranges are described by the AAMC as representing different proficiency levels on the target competencies.^6^

We used linear regression analysis to assess the relationship between SVI score and TI score. Analysis of variance (ANOVA) was used to determine the variation of mean TI score with the SVI subgroup score. Interrater reliability of TI for applicants who interviewed at more than one program was calculated using the intraclass coefficient.

This study was reviewed by the institutional review board at the primary site.

## RESULTS

Six ACGME-accredited EM residency programs participated in the study. Demographic data are listed in Table 1. A total of 344 applicants were assigned to a blinded interviewer. Seven were excluded due to prior knowledge of the applicant, and 16 were excluded as no SVI had been completed. This left 321 unique applicants for final analysis. Demographic data were available for 318 (Table 2) as some institutions blocked ERAS demographics.

SVI scores for the applicants ranged from 10–28 (mean = 20 ± 2.8). Interview scores ranged from 1–5 (mean = 3.4 ± 0.9). Linear regression analysis of the SVI score against the TI score demonstrated a small, positive linear correlation with an *r* coefficient of +0.13 (p = 0.02). When separating SVI scores into subgroups, this relationship between the SVI score and the TI score remained (p = 0.03) (Table 3).

Thirty-four applicants had interviews at more than one site (range 2–3 sites, mean 2.1). The intraclass coefficient of TI scores for these applicants was low (ICC = 0.023).

## DISCUSSION

Residency programs receive hundreds to thousands of medical student applications each year. Screening this volume of applications to decide which applicants to invite to interview can be daunting, and much of the process remains subjective. There have been many attempts at innovative approaches to standardization of the application process over the past several years. Most notably, this includes the SLOE, which is widely used by EM clerkship directors to provide grading transparency and standardization.^7^ Similarly, the AAMC has now developed the SVI as another tool for residency programs to help differentiate students in the competencies of interpersonal and communication skills and professionalism in a more standardized fashion prior to TI.

We found, not surprisingly, that there was a small, positive linear correlation between the SVI score and the TI score. This correlation remained across all SVI score subgroups. As the SVI score increased, the TI score increased as well. This suggests that, in many cases, the SVI may provide an estimate of an applicant’s performance on a TI. SVI and TI may be assessing the same qualities in applicants, such as verbal communication skills, emotional intelligence, teamwork and leadership, empathy and altruism, ethics, cultural competence, and conscientiousness.^1^,^2^ Although we found a positive correlation between the SVI and the TI, the *r* coefficient was low (*r* = +0.13). For every one point increase in SVI score, the TI increased by 0.04. This indicates that while the SVI may approximate the TI, it may not be a true replacement for a real interview.

While we have demonstrated through our analysis that the SVI may be a proxy for an interviewer assessing an applicant in a TI, it does not provide the applicant an opportunity to learn more about the residency program and determine their “fit.”^8^,^9^ In addition, many interview days are preceded by a pre-interview social event during which the applicants may freely interact with the residents without the formal constraints of the interview day.^10^ The uni-directional SVI format does not allow for this bi-directional matching process between the applicants and programs and for this reason is unlikely to ever fully replace the TI day.

## LIMITATIONS

Although this was a multicenter study that included a diverse representation of residency programs, only 321 of the 2901 applicants to EM residency programs during this application cycle were included for analysis. This may limit the overall generalizability of our findings. In addition, we did not use structured interviews. Each blinded traditional interviewer was allowed to ask the questions that he or she typically asks and conduct themselves during the interview process as they normally would, independent of the study. We felt that this would be more reflective of the real-world performance of the TI. However, not surprisingly, we found a low interrater reliability (intraclass correlation coefficient = 0.023) among a high number of interviewers (n = 50). This is an interesting result in and of itself, irregardless of the SVI. This may reflect a varied interview process at each of the different participating sites, making it difficult to compare TI scores from program to program. Lastly, we only included applicants who were randomly assigned to a blinded interviewer, which may have resulted in a sample bias.

## CONCLUSION

Our study suggests that there is a small, positive linear correlation between the Standard Video Interview and performance during the traditional interview. Future directions include determining which aspects of interview performance are assessable by both the SVI and the TI and which are uniquely measured by the TI alone.

## Figures and Tables

**Table 1:** Demographic data of residency programs and traditional interviewers.

Residency programs		
Number of programs	6	
University	5 (83%)	
Community	1 (17%)	
Northeast	3 (50%)	
South	2 (33%)	
West	1 (17%)	

Interviewers		Years of experience interviewing applicants (Range; Mean ± SD)

Number of interviewers	50	1–25; 5.8 ± 6.1
Position		
Chair	1 (2%)	8
Program Director	1 (2%)	15
Associate/Assistant Program Director	5 (10%)	3–20; 7.6 ± 7.1
Clerkship Director	1 (2%)	10
Core Faculty	10 (20%)	4–25; 12 ± 7.3
General Faculty	21 (42%)	1–20; 4 ± 4.2
Chief Resident	4 (8%)	1
Resident	7 (14%)	1–3; 1.9 ± 0.7

*SD*, standard deviation.

**Table 2:** Demographic data of residency applicants.

Demographic	N=318	Range	Median	Mean
Age		23–38	27	27.1 ± 2.4
Gender (n=312)				
Male	192 (61.5%)			
Female	120 (38.5%)			
Medical School				
Northeast	131 (41.2%)			
Central	50 (15.7%)			
South	92 (28.9%)			
West	33 (10.4%)			
International	12 (3.8%)			
US Private	122 (38.4%)			
US Public	158 (49.7%)			
Osteopathic	26 (8.2%)			
International	12 (3.8%)			
USMLE Step 1		195–272	235	235.5 ± 15.1
USMLE Step 2 CK		215–284	250	248.8 ± 13.5
USMLE Step 2 CS				
Pass	100%			
COMLEX Level 1		430–773	598	591.3 ± 85.5
COMLEX Level 2 CE		501–913	617	634.9 ± 110
COMLEX Level 2 PE				
Pass	100%			

*USMLE*, United States Medical Licensing Exam; *CK*, clinical knowledge; *CS*, clinical skills; *COMLEX*, Comprehensive Osteopathic Medical Licensing Exam; *CE*, cognitive evaluation; *PE*, performance evaluation.

**Table 3:** Relationship between the Standardized Video Interview score and traditional interview score by subgroup.

SVI Score Subgroup	N	Mean TI Score	P
6–11	1	3	0.03*
12–17	55	3.1 ± 0.9	
18–23	225	3.46 ± 0.9	
24–30	40	3.51 ± 0.9	

*SVI*, Standardized Video Interview; *TI*, traditional interview.

* p<0.05 denotes statistical significance.
